# Zinc and diabetes mellitus: understanding molecular mechanisms and clinical implications

**DOI:** 10.1186/s40199-015-0127-4

**Published:** 2015-09-17

**Authors:** Priyanga Ranasinghe, Shehani Pigera, Priyadarshani Galappatthy, Prasad Katulanda, Godwin R. Constantine

**Affiliations:** Department of Pharmacology, Faculty of Medicine, University of Colombo, Colombo, Sri Lanka; Diabetes Research Unit, Department of Clinical Medicine, Faculty of Medicine, University of Colombo, Colombo, Sri Lanka

## Abstract

**Background:**

Diabetes mellitus is a leading cause of morbidity and mortality worldwide. Studies have shown that Zinc has numerous beneficial effects in both type-1 and type-2 diabetes. We aim to evaluate the literature on the mechanisms and molecular level effects of Zinc on glycaemic control, β-cell function, pathogenesis of diabetes and its complications.

**Methods:**

A review of published studies reporting mechanisms of action of Zinc in diabetes was undertaken in PubMed and SciVerse Scopus medical databases using the following search terms in article title, abstract or keywords; (“Zinc” or “Zn”) and (“mechanism” or “mechanism of action” or “action” or “effect” or “pathogenesis” or “pathology” or “physiology” or “metabolism”) and (“diabetes” or “prediabetes” or “sugar” or “glucose” or “insulin”).

**Results:**

The literature search identified the following number of articles in the two databases; PubMed (*n* = 1799) and SciVerse Scopus (*n* = 1879). After removing duplicates the total number of articles included in the present review is 111. Our results show that Zinc plays an important role in β-cell function, insulin action, glucose homeostasis and the pathogenesis of diabetes and its complications.

**Conclusion:**

Numerous *in-vitro* and *in-vivo* studies have shown that Zinc has beneficial effects in both type-1 and type-2 diabetes. However further randomized double-blinded placebo-controlled clinical trials conducted for an adequate duration, are required to establish therapeutic safety in humans.

**Electronic supplementary material:**

The online version of this article (doi:10.1186/s40199-015-0127-4) contains supplementary material, which is available to authorized users.

## Introduction

Diabetes mellitus is a leading cause of morbidity and mortality worldwide, with an estimated 387 million adults being affected in year 2014, a figure which is expected to increase by nearly 40 % by year 2035 [1]. Ninety to ninety five percent of those with the disease have type-2 diabetes. In a patho-physiologic sense, type-2 diabetes is a multi-organ, multi-factorial condition characterized primarily by insulin resistance, hyper insulinaemia and β-cell dysfunction, which ultimately leads to β-cell failure [2]. Type-1 diabetes has historically been most prevalent in populations of European origin, and the latest edition of the Diabetes Atlas estimates that 490,100 children below the age of 15 years are living with type-1 diabetes [1]. Currently 77 % of those with diabetes live in low- and middle-income countries of the African, Asian, and South American regions [1, 3, 4]. In 2014, diabetes was responsible for 4.9 million deaths worldwide and at least US$ 612 billion in global healthcare expenditures (11 % of the total global healthcare expenditures in adults) [1]. Recent cost estimates, include those for Brazil (US$ 3.9 billion), Argentina (US$ 0.8 billion) and Mexico (US$ 2.0 billion) [5]. Each of these is an annual figure and is rising as diabetes prevalence increases. Overall, direct health care costs of diabetes ranges from 2.5 to 15 % annual health care budgets, depending on local diabetes prevalence and the sophistication of the treatment available [5]. Diabetes is also associated with a host of potentially disabling macro- and micro-vascular complications. Hence, there is also a much larger burden in the form of lost productivity as a result of restricted daily activity. This rapidly increasing prevalence is attributable to population growth, aging, urbanization, unhealthy dietary habits, increasing prevalence of obesity and physical inactivity [6].

Although comprehensive diabetes management guidelines are readily available, even in developed countries like the US 30–50 % adults with diabetes do not meet individualized targets for glycaemic, blood pressure, or lipid control [7]. Reasons for failure to achieve glycaemic control includes the progression of underlying β-cell dysfunction, incomplete adherence to treatment (often due to adverse effects of medication) and reluctance of clinicians to intensify therapy [8]. Anti-diabetic agents currently in use can directly or indirectly enhance the functioning of β-cells. However, reducing the decline and the eventual failure of β-cells is crucial in preventing type-2 diabetes in those at risk and halting disease progression in the affected patients [8]. The increasing worldwide prevalence of type-2 diabetes and the progressive loss of metabolic control in patients are clear demonstrations that the current therapeutic strategies aimed at protecting the β-cells are largely inadequate. Hence there is an urgent need for anti-diabetic agents targeting the intimate mechanisms of β-cell damage and optimizing its function at cellular level.

Insulin, is stored as a hexamer containing two Zinc ions in β-cells of the pancreas and released into the portal venous system at the time of β-cell de-granulation [9]. *In-vitro* and *in-vivo* studies in animals and humans have shown that Zinc has numerous beneficial effects in both type-1 and type-2 diabetes [10–14]. A recent meta-analysis confirmed these findings, and concluded that Zinc supplementation in patients with diabetes improves glycaemic control and promotes healthy lipid parameters [15]. Hence, it is evident that Zinc has a promising potential as a novel therapeutic agent in diabetes. Studies have also shown that diabetes is commonly accompanied by hypozincemia and hyperzincuria [16, 17]. Furthermore the high prevalence of Zinc deficiency in developing countries could be contributing towards driving the current diabetes epidemic encountered by them [4, 18]. Numerous research studies have been conducted to clarify the molecular mechanisms underlying the action of Zinc in diabetes. Understanding the molecular mechanisms of action of Zinc will help to further develop targeted therapy and guide future research. The present study aims to systematically evaluate the literature on the mechanisms and molecular level effects of Zinc on glycaemic control, β-cell function, pathogenesis of diabetes and its complications.

## Methods

A systematic review of published studies reporting mechanisms of action of Zinc in diabetes was undertaken in accordance with the Preferred Reporting Items for Systematic reviews and Meta-Analyses (PRISMA) statement (Additional file 1).

### Search strategy

A comprehensive search of the literature was conducted in the PubMed® (U.S. National Library of Medicine, USA) and SciVerse Scopus® (Elsevier Properties S.A, USA) databases for studies published before 31st July 2015. During the first stage the above databases were searched using the following search terms in article title, abstract or keywords; (“Zinc” or “Zn”) and (“mechanism” or “mechanism of action” or “action” or “effect” or “pathogenesis” or “pathology” or “physiology” or “metabolism”) and (“diabetes” or “prediabetes” or “sugar” or “glucose” or “insulin”).

In the second stage the total hits obtained from searching these three databases were pooled together and duplicates were removed. This was followed by screening of the retrieved articles by reading the article title in the third and abstracts in fourth stage. In the fifth stage individual manuscripts were screened, and those not satisfying inclusion criteria (given below) were excluded. This search process was conducted independently by two reviewers (PR and SP) and the final group of articles to be included in the review was determined after an iterative consensus process.

### Inclusion/exclusion criteria and data extraction

The following inclusion criteria were used; a) *In-vitro* or *in-vivo* studies reporting effect of Zinc on diabetes/pre-diabetes, evaluating effects of Zinc on sugar/glucose, insulin and/or related metabolic parameters, evaluating the effects of Zinc on pathogenesis and/or complication of diabetes b) Published in English, or with detailed summaries in English and c) Peer-reviewed fully published research papers. Conference proceedings, editorials, commentaries, review articles and book chapters/book reviews were excluded.

Data were extracted from the included studies by one reviewer using a standardized form and checked for accuracy by a second reviewer. The data extracted from each study were: a) study details (lead author, year published/year of survey, type of study—*In-vitro/In-vivo*), b) methods (study design, sample size, duration) and c) mechanism of action data. Discrepancies in the extracted data were resolved by discussion, with involvement of a third reviewer when necessary.

## Results

**Fig. 1:**
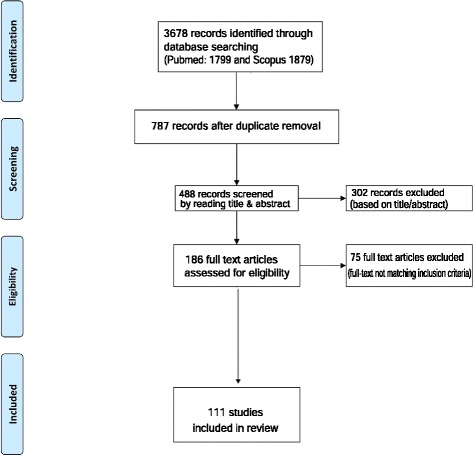
Summarized search strategy

The literature search using the above search criteria identified the following number of articles in the two databases; Medline® (*n* = 1799) and SciVerse Scopus® (*n* = 1879). After removing duplicates the total number of articles included in the present review is 111. The search strategy is summarized in Fig. 1.

### Anti-oxidant properties

Anti-oxidant properties of Zinc have been evaluated *in-vitro*, as well as *in-vivo* animal and human studies. Hypozincaemia and hyperzincuria is known to be present in patients with both type-1 and type-2 diabetes, and Zinc supplementation is known to be helpful in restoring plasma Zinc levels to normal [11, 19–21]. Plasma Thiobarbituric acid reactive substances (TBARS), a marker of oxidative stress is high in both type-1 and type-2 diabetic patients, and significantly decreased by supplementation of Zinc 30 mg/day for 3–6 months [11, 19]. Selenium-dependent glutathione peroxidase (Se-GPx), an anti-oxidant enzyme was also low at baseline in patients with type-1 diabetes, and was normalized after Zinc supplementation [11]. However, no significant difference in antioxidant metallo-enzyme activity was observed in patients with type-2 diabetes [19]. Plasma Zinc levels are known to be negatively correlated with the TBARS levels in both obese and non-obese patients with type-2 diabetes [22].

In animal models of insulin resistance, Zinc supplementation is known to enhance insulin sensitivity and antioxidant status [23, 24]. Furthermore, in diabetes induced animal models anti-oxidant enzymes catalase, GPx and super-oxide dismutase (SOD) are decreased than in normal animals [25–27]. Zinc supplementation in these animals restored the enzyme activity and increased glutathione synthesis [25–27]. Plasma malondialdehyde (MDA) levels, an index of lipid peroxidation was increased in diabetic animals and significantly decreased following Zinc supplementation [26, 28]. When rats were simultaneously treated with a single injection of alloxan and ZnCl_2_, the increase in blood glucose concentration induced by alloxan was significantly reduced at 24, 48, and 72 h post-treatment with ZnCl_2_ [29]. ZnCl_2_ injection also increased the retinal, pancreatic, and liver glutathione and reduced the TBARS content in comparison with the alloxan-treated group [29]. Zinc supplementation or injection is also known to cause a significant induction of anti-oxidant metallothionein (MT) protein synthesis in the pancreatic islets, kidneys, liver and heart of diabetes-induced animals [27, 28, 30–34].

A significant increase in cardiac morphological impairment, fibrosis, and dysfunction is seen in diabetic mice, which is reversed by Zinc [33]. Cultured cardiac cells that were directly exposed to high levels of glucose (HG) and free fatty acid (FFA), treatment that mimics diabetes, resulted in reduced cell survival rate, which was reversed by Zinc [33]. Furthermore, when MT expression was silenced with the use of MT small-interfering RNA, the preventive effect of pretreatment with Zinc was abolished [33]. Hence, the prevention of diabetic cardiomyopathy by Zinc supplementation is predominantly mediated by an increase in cardiac MT and the resultant anti-oxidant effects [33, 35]. Diabetes-induced renal oxidative damage, inflammation and up-regulated expression of pro-fibrosis mediators were also markedly attenuated by Zinc supplementation, mediated via the expression of MT [27, 36]. *In-vivo* studies in diabetes induced rats have shown that Zinc has a protective effect against diabetes induced peripheral nerve damage by stimulating MT synthesis and reducing oxidative stress [37].

Diabetes induced a significant increase in aortic oxidative damage, inflammation, and remodeling in mice (increased fibrosis and wall thickness) [38]. Zinc treatment of these diabetic mice completely prevented the above pathogenic changes in the aorta, and also significantly up-regulated the expression and function of nuclear factor (erythroid-derived 2)-like 2 (Nrf2), a pivotal regulator of anti-oxidative mechanisms, and the expression of MT [38]. The same up-regulation of Nrf2 by Zinc has been demonstrated in human renal tubule cells *in-vitro* and mouse kidney *in-vivo* under the diabetic conditions [39]. In cultured renal tubular epithelial cells (NRK-52E), Zinc supplementation inhibited high glucose induced cell apoptosis by attenuating reactive oxygen species production, facilitated via Nrf2 up-regulation [40]. In postmenopausal women with type-2 diabetes Zinc supplementation increases TNF-α gene expression, suggesting a close interaction between Zinc homeostasis, oxidative stress and inflammation [41].

### Effects on carbohydrate and lipid metabolism

Zinc is known to stimulate glycolysis and inhibit gluconeogenesis, an effect that is not overcome by the presence of glucagon [42–44]. *In-vitro* studies have demonstrated that Zinc increases the activity of glycolytic enzymes, phosphofructokinase (PFK) and pyruvate kinase (PK) in a concentration- and time-dependent manner [45, 46]. Lactate production which reflects the PFK activity, was increased by Zinc [46]. However, the effects of Zinc and insulin were not additive and Zinc pretreatment prevented the stimulation of glycolytic enzymes by insulin. Furthermore, in Zinc-treated cells a progressive activation of ERK2/MAPK1 (Mitogen-activated protein kinase-1) was observed [45]. However, a recent *in-vitro* study using hepatocytes has shown that at sub-lethal concentrations, ZnO nanoparticles increase both gluconeogenesis and glycogenolysis, contradicting the finding from the earlier studies given above [47].

Zinc is also known to be a concentration dependent reversible inhibitor of α-glucosidase activity in the intestines [48, 49]. Zinc binding to α-glucosidase directly induced tertiary structural changes, however the concentration required to induce structural changes was greater than that required to achieve inhibition [49]. In skeletal muscles Zinc-α2-glycoprotein stimulates the phosphorylation of AMP-activated protein kinase (AMPK*α*) and increases cellular GLUT4 protein [50]. This increase in the expression of the GLUT4 has also been observed in adipose tissue with resultant increase in glucose uptake [44, 51, 52]. Studies have also shown that whole body as well as adipose tissue specific insulin sensitivity was positively associated with Zinc-α2-glycoprotein expression in subcutaneous adipose tissue and that obesity related decrease in Zinc-α2-glycoprotein is selectively present in subcutaneous but not in visceral adipose tissue [53]. However, two recent studies have indicated that Zinc-α2-glycoprotein functions to inhibit insulin signaling and, in consequence, insulin-induced glucose uptake in adipocytes, whereas, Zinc Finger Protein 407 (ZFP407) is known to stimulate GLUT4 mRNA transcription in adipocytes, facilitating insulin-stimulated glucose uptake via GLUT4 [54, 55].

Zinc also enhanced glucose transport in adipocytes in a dose dependant manner, independent of insulin [43, 56–58]. Zinc was observed to stimulate the phosphorylation of the IR-ß (Insulin Receptor) subunit [52, 56]. Zinc also inhibits Glycogen synthase kinase-3 (GSK-3β), which is a phosphorylating and an inactivating agent of glycogen synthase, with resultant increase in glycogen synthesis [57]. Furthermore, Zinc-α2-glycoproteins increase the expression of the lipolytic enzymes, adipose triglyceride lipase and hormone-sensitive lipase in the white adipose tissue [51]. Incubation of adipocytes with Zinc, augmented lipogenesis and this lipogenic effect was 80 % of maximum insulin stimulation [43, 59]. In isolated rat liver membrane cells, Zinc alone stimulated lipogenesis in a dose-related manner [60].

Treatment of 3 T3-L1 adipocytes with Zinc-chelated vitamin C (ZnC) has been shown to promote adipogenesis, characterized by increased glycerol-3-phosphate dehydrogenase activity and intracellular lipid accumulation in 3 T3-L1 cells, associated with a pronounced up-regulation of the expression of glucose transporter type 4 (GLUT4) [61]. ZnC further increased the expression of peroxisome proliferator-activated receptor gamma (PPARγ) and CCAAT/enhancer-binding protein alpha, the key transcription factors of adipogenesis [61]. A decrease in insulin receptor binding has been observed in Zinc deficient rat adipocytes and addition of Zinc stimulates insulin binding in a dose-dependent manner [60, 62].

### Islet cell function

*In-vitro* environments increasing the extracellular Zinc concentration is known to increase the free insulin concentration in the immediate vicinity of β-cells, mediated by enhanced Zinc-insulin dissociation [63]. However, glucose stimulated insulin secretion in β-cells of the pancreas is inhibited by Zinc, suggesting that co-secreted Zinc acts in an autocrine inhibitory modulator [64, 65]. In pancreatic β-cells of mice and clonal HIT-T15 β-cells, KCl and glucose induced an increase in free cytosolic Zinc levels, which facilitated the processing and/or storage of insulin [66, 67]. This is facilitated by an increase in the expression of cellular Zinc importers (Slc39a6, Slc39a7, and Slc39a8). However, chronic increase in cytosolic Zinc levels following sustained hyperglycemia, as in diabetes may contribute towards β-cell dysfunction and death [66].

Human Islet Amyloid Polypeptide (hIAPP) (a polypeptide hormone secreted from pancreatic β-cells in response to glucose) and is cleared by the peptidases in the kidney. hIAPP is known to aggregate in the pancreas to form dense, insoluble extracellular fibrillar deposit, causing β-cell destruction in type-2 diabetes [68]. Zinc, significantly inhibits hIAPP amyloid fibrillogenesis at concentrations similar to those found *in-vivo* extracellular environments [68]. This probably explains the linkage between the mutations of SLC30A8 zinc transporter (Zinc Transporter 8 [ZnT8]), which transports Zinc into the secretory granules, and type-2 diabetes. In human pancreatic islet cells, ZnT8 is the key protein responsible for both intracellular Zinc accumulation in insulin-containing vesicles and regulation of insulin secretion [69–73]. ZnT8 down regulated cells show reduced insulin content and decreased insulin secretion in response to hyperglycemic stimuli [74]. However, absence of ZnT8 expression did not alter rates of insulin biosynthesis, insulin content and glucose metabolism, but contributed to the packaging efficiency of stored insulin [75–77]. When ZnT8 absent mice were fed a control diet, glucose tolerance and insulin sensitivity were normal. However, after high-fat diet feeding, these mice became glucose intolerant or diabetic, and islets became less responsive to glucose [75]. ZnT8 is down regulated on exposure to metabolic stress associated with diabetic and pre-diabetic states, suggesting that it might further contribute to progression of type-2 diabetes [78, 79]. In β-cell specific SLC30A8 deficiency (ZnT8 knockout mice) a low peripheral blood insulin levels was observed, due to a substantial amount of the insulin being degraded during its first passage through the liver [80]. This is possibly due to the low level of Zinc in the portal circulation co-secreted by β-cells, due to the absence of ZnT8 (reducing uptake of Zinc by β-cells), which leads to augmented hepatic insulin clearance. The ZnT8 is also downregulated in response to exposure of pancreatic β-cells to hypoxia, resulting in lowered cytosolic Zn^2+^ concentrations [81].

Pancreatic islet cells harvested from rats conditioned under intermittent hypoxia showed a significant reduction in Zinc Influx Transporter 8 (ZIP8) expression in the β-cell membrane, with resultant reduction in cellular Zinc concentration and insulin production [82]. ZIP6 and ZIP7 function as two important zinc influx transporters to regulate cytosolic Zinc concentrations and insulin secretion in β-cells and ZIP-6 is also capable of directly interacting with GLP-1R to facilitate the protective effect of GLP-1 on β-cell survival [83]. Zip4 protein is located in human pancreatic β-cells, is important for the accumulation of Zinc in the cytosol and granules of β-cells [84]. Other Zinc transporters like ZnT3 and ZIP7 might also play a role in insulin secretion and glucose metabolism [85, 86].

L-type voltage-gated Ca^2+^ channels and TRMP3 (transient receptor potential cation channel subfamily M member 3) are also in part responsible for Zinc transport into β-cells, which is also dependent upon the metabolic status of the cell [87, 88]. Culture of rat pancreatic islets in either low or high vs. intermediate glucose concentrations triggers early mitochondrial oxidative stress and late β-cell apoptosis with loss of glucose stimulated insulin secretion [89]. ZnCl_2_ reduces mitochondrial oxidative stress and rat β-cell apoptosis under these culture conditions [89]. ZnO nanoparticles at dose of 70 ng/mL improved viability and function of pancreatic islets, by reducing oxidative stress and preventing cells from entering the apoptotic phase [90]. It is well known that Reactive Oxygen Species (ROS) can cause pancreatic β-cell death. This occurs due to the activation of Transient Receptor Potential Melastatin2 (TRPM2) channels by ROS. TRPM2 causes Ca^2+^ influx into the β-cells causing release of lysosomal Zinc, which results in β-cell death [91]. The tumor suppressor gene ST18 is a neural Zinc finger transcription factor, expressed in pancreatic β-cells and is known to impair insulin secretion, induce β-cell apoptosis and curtail β-cell replication [92].

In glucagon producing α-cells of the pancreas Zinc accumulates under low and high glucose conditions through both Ca^2+^channels and other Zinc transporting mechanisms, and the intracellular Zinc inhibits glucagon secretion [93]. Furthermore during hypoglycemia the principal signal that initiates glucagon secretion could be the detection by α-cells of a sudden decrease in Zinc paralleling the fall in insulin in the islet peri-portal circulation and this drop in concentration of Zinc, closes α-cell ion channels, promoting entry of calcium which stimulates glucagon secretion [94, 95].

### Insulin-mimetic compounds

Zinc ions and its’ complexes, have shown insulin-like action both *in-vitro* and *in-vivo* experiments (Table 1) [96–117]. *In-vitro*, Zinc complexes inhibit the release of FFA from cultured rat adipocytes [96–104, 106, 108, 110–112, 114, 116]. In cultured 3 T3-L1 adipocytes these Zinc complexes activates the insulin signaling cascade through Akt/PKB (protein-kinase B) phosphorylation resulting in GLUT4 translocation to the plasma membrane and enhanced cellular glucose uptake [105, 107, 113]. *In-vivo* type 2 diabetic KK-Ay mice, these complexes have demonstrated an ability to reduce blood glucose, HbA1c, triglycerides and total cholesterol [98, 100, 102, 104, 110–112, 116, 117]. They are also known to improve the glucose tolerance as demonstrated by Oral Glucose Tolerance Testing (OGTT) [96, 99, 103, 110, 114, 117].

**Table 1:** Summary of Zinc Insulin-mimetic compounds

Author, year	Zinc complexes evaluated	Study design	Main findings	Conclusion
Adachi, et al. 2004 [100]	Bis(maltolato)-zinc(II) [Zn(ma)_2_]	*In vitro—*isolated rat adipocytes	*In-vitro—*Inhibitory activity on FFA release observed with Zn(alx)_2_, Zn(ma)_2_, Zn(ema)_2_ and Zn(3 hp)_2_; Zn(alx)_2_ exhibited the highest; Zn(alx)_2_ and Zn(ma)_2_ induced a concentration dependent increase in glucose uptake	Significant insulin-mimetic properties were exhibited by Zn(alx)_2_
	Bis(allixinato)-zinc(II) [Zn(alx)_2_]	*In-vivo*—Type 2 diabetic KK-A^y^ mice (i.p. injections for 14 days); Zn(alx)_2_ was compared with that of Zn(ma)_2_		
	Bis(3-hydroxy-4-pyronato)-zinc(II) [Zn(3 hp)_2_]			
			In-vivo—Both complexes reduced BG, TG, leptin & insulin; HbA1c was lower with Zn(alx)_2_ > Zn(ma)_2_	
	Ethyl maltol-zinc(II) [Zn(ema)_2_]			
	Kojic acid-zinc(II) [Zn(ka)_2_]			
Adachi, et al. 2007 [104]	Zinc(II)-N-acetyl-L-cysteine [Zn(NAC)]	*In-vitro*—Isolated rat adipocytes	*In-vitro*—A dose-dependent inhibitory effect on FFA release	Zn(NAC) improves insulin resistance and glucose tolerance; Low bioavailability with oral administration (22.3 %)
		*In-vivo*—Type 2 diabetic KK-A^y^ mice (i.p. injections for 28 days)	*In-vivo*—BG lowered to normal; BW, serum TG and FFA levels unchanged; TC reduced; Serum insulin and HbA1c reduced	
Basuki, et al. 2007 [105]	Bis(1-oxy-2-pyridine-thiolato)-zinc(II) [Zn(opt)_2_]	*In-vitro*—3 T3-L1 adipocytes	Zn(opt)_2_ induced concentration- and time-dependent Akt/PKB (protein-kinase B) phosphorylation and increased GLUT-4 levels in cell membrane	Zn(opt)_2_ exhibited insulin-mimetic activity by activating insulin signalling cascade through Akt/PKB phosphorylation resulting in GLUT4 translocation
	Bis(picolinato)-zinc(II) [Zn(pa)_2_]			
	Bis(aspirinato)-zinc(II) [Zn(asp)_2_]			
	Bis(1-oxy-2-pyridonato)-zinc(II) [Zn(opd)_2_]			
Fujimoto, et al. 2013 [116]	Di(2-selenopyridine-N-oxidato)zinc(II) [ZPS]	*In-vitro*—Isolated rat adipocytes	*In-vitro*—A dose-dependent inhibitory effect on FFA release	ZPS exhibits anti-diabetic activity, even at low doses.
		*In-vivo*—Type 2 diabetic KK-A^y^ mice (Oral for 28 days)	*In-vivo*—BG and HbA1c reduced; TG, TC, insulin, leptin and adiponectin levels unchanged	
Kadowaki, et al. 2013 [117]	Zinc-3,4-heptanedione-bis(N^4^-methylthiosemicarbazonato) (Zn-HTSM)	*In-vivo*—Type 2 diabetic KK-A^y^ mice (Oral for 14 days)	*In-vivo*—BG lowered to normal; Serum leptin reduced; improved glucose tolerance with OGTT; Serum insulin and adiponectin unchanged	Zn-HTMS has anti-diabetic activity and also acts on leptin metabolism
Karmaker, et al. 2009 [110]	Zinc(II)-Poly-γ-glutamic acid [Zn(γ-pga)]	*In-vitro*—Isolated rat adipocytes	*In-vitro*—A dose-dependent inhibitory effect on FFA release and enhanced glucose uptake	Significant insulin-mimetic properties were exhibited by Zn(γ-pga) complex
		*In-vivo*—Type 2 diabetic KK-A^y^ mice (Oral for 30 days)	*In-vivo*—BG lowered to normal; HbA1c and insulin reduced; improved glucose tolerance with OGTT; TC and TG unchanged;	
Kojima, et al. 2005 [102]	Zinc(II)-6-ethylpicolinate [Zn(6epa)_2_]	*In-vitro*—Isolated rat adipocytes	*In-vitro*—A dose-dependent inhibitory effect on FFA release	Significant insulin-mimetic properties were exhibited by Zn(6epa)_2_
		*In-vivo*—Type 2 diabetic KK-A^y^ mice (i.p. injections for 14 days)	*In-vivo*—BG lowered to normal; serum TG and TC unchanged; TC reduced; Serum HbA1c reduced	
Matsumoto, et al. 2011 [111]	Zn(II)-ascorbic acid [Zn(VC)_2_]	*In-vitro*—Isolated rat adipocytes	*In-vitro—*Inhibitory activity on FFA release; highest activity Zn(VC)_2_	A Zn(II) complex with VU or VC showed preventive effects on metabolic syndrome in Fructose Fed Rats
	Zn(II)-methylmethionine sulfonium [Zn(VU)_2_)]	*In-vivo*—Fructose fed rats (oral, 4 weeks)	*In-vivo*—Zn(VU)_2_ Significantly reduced mesenteric adipocytes and BG; TC and TG unchanged;	
	Zn(II)-L-carnitine [Zn(Car)_2_]			
Moniz, et al. 2011 [112]	Zinc(II) complexes of 3-hydroxy-4-pyridinones	*In-vitro*—Isolated rat adipocytes	*In-vitro—*Inhibitory activity on FFA release	Zinc(II)-3-hydroxy-4-pyridinones showed insulin-mimetic properties
		*In-vivo*—STZ induced diabetic rats (i.p injections for 33 hrs)	*In-vivo*—BG lowered	
Naito, et al. 2011 [113]	Di(hinokitiolato)-zinc(II) [Zn(hnk)_2_]	*In-vitro*—3 T3-L1 adipocytes	Zn(hnk)_2_ induced dose dependant AKt/PKB phosphorylation, stimulated GSK3β in a dose-dependent manner and enhanced glucose uptake	Zn(hnk)_2_ showed insulin-mimetic properties by inducing insulin signalling pathways
	Di(tropolonato)-zinc(II) [Zn(trp)_2_]			
Nakayama, et al. 2008 [107]	Bis(allixinato)-zinc(II) [Zn(alx)_2_]	*In-vitro*—3 T3-L1 adipocytes	Both complexes induced concentration- and time-dependent Akt/PKB (protein-kinase B) phosphorylation and increased GLUT-4 levels in cell membrane; They also inhibited FFA release	Zn(alx)_2_ and Zn(tanm)_2_ activated the Akt/PKB-mediated insulin-signalling pathway and improved utilization and lipid metabolism
	Bis(thioallixin-N-methyl)-zinc(II) [Zn(tanm)_2_]			
Nishide, et al. 2008 [108]	Bis(pyrrole-2-carboxylato)-zinc(II) [Zn(pc)_2_]	*In-vitro*—Isolated rat adipocytes	*In-vitro—*Inhibitory activity on FFA release seen with all complexes; Zn(ta)_2_ showed highest activity	Significant insulin-mimetic properties were exhibited by Zn(ta)_2_
	Bis(α-furonic acidato)-zinc(II) [Zn(fa)_2_]			
	Bis(thiophene-2-carboxylato)-zinc(II) [Zn(tc)_2_]			
	Bis(thiophene-2-acetato)-zinc(II) [Zn(ta)_2_]			
Rasheed, et al. 2008 [109]	Zinc (II) glibrnclamide [Zn(II)–GBA]	*In-vivo—*Alloxan treated diabetic rabbits (oral, single dose)	The Zn(II)—GBA showed a faster on set of action with prolonged duration compared to the standard drug(GBA)	The Zn(II)—GBA complex showed significant hypoglycaemic activity
Ueda, et al. 2002 [98]	Zinc(II)-2-picolinamide [Zn(pa-a)_2_]	*In-vitro*—Isolated rat adipocytes	*In-vitro*—A dose-dependent inhibitory effect on FFA release	Significant insulin-mimetic properties were exhibited by Zn(pa-a)^2^ and
	Zinc(II)-6-methyl-2-picolinmethylamide [Zn(6mpa-ma)_2_]	*In-vivo*—Type 2 diabetic KK-A^y^ mice (i.p. injections for 14 days)	*In-vivo*—BG and HbA1c lowered; TC unchanged;	Zn(6mpa-ma)_2_
Vijayaraghavan, et al. 2012 [115]	Zinc-3-hydroxy flavone [Zn-flavonol]	*In-vivo*—STZ induced diabetic rats (Oral, 30 days)	At 5, 10, 20 and 50 mg/kg/day, Zn-flavonol complex exhibited significant hypoglycaemic activity; HbA1c, glucose and insulin levels were restored to near normal	Zn-flavonol complex has significant anti-hyperglycemic activity
Yoshikawa, et al. 2001 [96]	Bis(maltolato)-zinc(II) [Zn(ma)_2_]	*In-vitro*—Isolated rat adipocytes	*In-vitro*—A dose-dependent inhibitory effect on FFA release; Combination of insulin and Zn(ma)_2_ further enhanced inhibitory effect than insulin or Zn(ma)_2_ alone	Zn(ma)_2_ improves insulin resistance and glucose tolerance
		*In-vivo*—Type 2 diabetic KK-A^y^ mice (i.p. injections for 14 days)		
			*In-vivo*—BG lowered to normal; serum TG and insulin reduced; FFA unchanged	
Yoshikawa, et al. 2001 [97]	Zinc (II) complexes of α-amino acids (L- and D- Asn, Pro, Thr, Val, Gly, Asp, Ala, Gln and His)	*In-vitro*—Isolated rat adipocytes	*In-vitro*—Only Zinc(II) complexes with lower over-all stability constants showed insulin-mimetic activity	There is an interrelationship between the stability constants and the insulin-mimetic activity of zinc(II) complexes
		*In-vivo*—Type 2 diabetic KK-A^y^ mice (i.p. injections for 14 days) (Only [Zn(L-Thr)_2_(H_2_O)_2_])		
			*In-vivo*—BG lowered to normal; improved glucose tolerance with OGTT	
Yoshikawa, et al. 2003 [99]	Bis(l-carnitinato)-zinc(II) [Zn(car)_2_]	*In-vitro*—Isolated rat adipocytes	*In-vitro*—A dose-dependent inhibitory effect on FFA release	Zn(car)_2_ improves insulin resistance and glucose tolerance
		*In-vivo*—Type 2 diabetic KK-A^y^ mice (oral for 16 days)	*In-vivo*—BG lowered; improved glucose tolerance with OGTT	
Yoshikawa, et al. 2004 [101]	Bis(picolinato)-zinc(II) [Zn(pa)_2_]	*In-vitro*—Isolated rat adipocytes	*In-vitro*—All 3 complexes inhibited FFA release and increased GLUT 4 levels	The complexes exhibited insulin-mimetic activity by activating insulin signalling and enhancing GLUT4 translocation
	Bis(maltolato)-zinc(II) [Zn(ma)_2_]			
	Bis(threoninato)-zinc(II) [ZT]			
Yoshikawa, et al. 2005 [103]	Zinc-2-aminomethyl-pyridine [Zn(2-ampy)_2_]	*In-vitro*—Isolated rat adipocytes	*In-vitro*—All 3 complexes inhibited FFA release [Zn(2-ampy)_2_ and Zn(1,5,9-TN) > Zn(1,5,8,12-TD)]	Zn(2-ampy)_2_ improves insulin resistance and glucose tolerance
	Zinc-1,5,9-Triazanonane [Zn(1,5,9-TN)]	*In-vivo*—Type 2 diabetic KK-A^y^ mice (i.p. injections for 14 days) (Zn(2-ampy)_2_ only)	*In-vivo*—BG and HbA1c lowered; improved glucose tolerance with OGTT;	
	Zinc-1,5,8,12-tetraazadodecane [Zn(1,5,8,12-TD)]			
Yoshikawa, et al. 2007 [107]	Zinc dimethyldithiocarbamic acid [Zn(dmd)_2_]	*In-vitro*—Isolated rat adipocytes	*In-vitro*—Zn(pdc)_2_ was most effective in inhibiting FFA and enhancing glucose-uptake	Zn(pdc)_2_ complex improves hyperglycemia and insulin resistance
	Zinc diethyldithiocarbamic acid [Zn(ded)_2_]	*In-vivo*—Type 2 diabetic KK-A^y^ mice (oral for 25 days) (Zn(pdc)_2_ only)	*In-vivo*—BG, insulin, HbA1c, TG, leptin and systolic BP reduced;	
	Zinc pyrrolidine-N-dithiocarbamic acid [Zn(pdc)_2_]			
	Zinc N-ethyl-N-phenyldithiocarbamate [Zn(epd)_2_]			
Yoshikawa, et al. 2011 [114]	Bis(aspirinato)-zinc(II) [Zn(asp)_2_]	*In-vitro*—Isolated rat adipocytes	*In-vitro*—No effect	Zn(asp)_2_ improves insulin resistance and glucose tolerance
		*In-vivo*—Type 2 diabetic KK-A^y^ mice (i.p. injections for 14 days and oral for 24 days)	*In-vivo*—BG lowered; improved glucose tolerance with OGTT;	

*BG* blood glucose; *BP* blood pressure; *FFA* free fatty acid; *GBA* glibenclamide; *GLUT* glucose transporter; *OGTT* oral glucose tolerance test; *TC* total cholesterol; *TG* triglycerides

In addition to Zinc containing complexes, Zinc Oxide nanoparticles (ZnO) are known to posses anti-diabetic activity. ZnO nanoparticles induce a significant reduction in blood glucose, elevates serum insulin levels and glucokinase activity, whilst stimulating a higher expression of insulin, insulin receptor, GLUT-2 and glucokinase genes in STZ induced (Type-1) diabetic rats [118]. Blood glucose level is also reduced in Type-2 diabetic rats administered ZnO nanoparticles, with improved glucose tolerance and a 70 % increase in serum insulin levels [119]. In addition a significant lowering of circulating triglycerides and free fatty acids was also observed suggesting a beneficial effect of ZnO on lipid metabolism [119].

### Other effects

In STZ-diabetes induced mice, Zinc supplementation has shown to increase the serum leptin concentration [120]. However, this finding has been contradicted by several other studies [100, 106, 121]. Hence, although it is evident that Zinc has an effect on leptin levels, the exact relationship and mechanisms are yet to be determined. *In-vitro*, ZnCl_2_ is known to stimulate the trans-differentiation of human heptoma (HpG2) cells into pancreatic-like cells, with increased expression of amylase and insulin mRNAs over 1000 and 10 000 fold respectively [122]. Zinc is also known to activate C-peptide, with resultant increased energy utilization in red cells leading to release of ATP, which in turn stimulates NO production in platelets and endothelium causing reduction in platelet activity [123].

Zinc supplementation has been shown to prevent bone loss in chronic T1DM rats by stimulating expression of the mineralizing phenotype in osteoblasts and reducing expression of the resorptive phenotype in osteoclasts, achieved by osteocalcin up-regulation and RANKL, OPG, COL1A, and MMP-9 protein down-regulation [124]. Furthermore, *in-vitro* Zinc inhibited advanced glycation end product (AGE)-induced MC3T3-E1 cell (mouse osteoblasts) apoptosis by attenuating the production of reactive oxygen species, inhibiting caspase-3 and caspase-9 activation, and inhibiting the release of cytochrome c from between the mitochondria and the cytosol [125]. Zinc significantly inhibited AGE formation of albumin *in-vitro* and reduced secondary and tertiary structural modifications of albumin and may have in controlling/preventing AGEs-mediated diabetic pathological conditions *in vivo* [126, 127].

## Discussion

Numerous *in-vitro* and *in-vivo* studies have shown that Zinc has beneficial effects in both type-1 and type-2 diabetes. A finding which has been confirmed by a recent meta-analysis, where Zinc supplementation resulted in improved glycaemic control [15]. It is evident from the findings of the present systematic review, that Zinc plays an important role in β-cell function, insulin action, glucose homeostasis and the pathogenesis of diabetes and its complications.

Our results clearly show that Zinc has anti-oxidant properties and that Zinc supplementation reduces oxidative stress. Zinc supplementation enhances the activity and levels of key anti-oxidant enzymes and proteins, whilst significantly reducing lipid peroxidation. Some of these effects are brought on by interactions at nuclear level, by stimulation of nuclear factors like Nrf2 [38]. It is well known that oxidative stress is high in both type-1 and type-2 diabetic patients, as evident by elevated TBARS levels in the plasma [11, 19]. This contributes towards further β-cell dysfunction, with resultant deterioration of glycaemic control. Oxidative stress also plays a pivotal role in the pathogenesis of both micro- and macro-vascular complications of diabetes [128]. The resultant increase in the production of ROSs is responsible for the activation of five major pathways involved in the pathogenesis of diabetes complications [128]. Increased formation of AGEs, and the increased expression of the receptor for AGEs and its activating ligands is one such primary pathway responsible for the pathogenesis of diabetes related complications [128]. Both *in-vitro* and *in-vivo* Zinc significantly inhibited the formation of AGEs. Hence, by alleviating the oxidative stress associated with diabetes and by reducing the formation of AGEs, Zinc supplementation could delay the progression of diabetes and also delay/prevent the numerous micro- and macro-vascular complications associated with diabetes.

Furthermore, our results show that Zinc plays an important role in glucose and lipid metabolism. Zinc reduces glucose absorption and synthesis, whilst promoting glucose metabolism and storage. This is primarily via the enhanced activity of key enzymes involved in these metabolic processes, such as α-glucosidase, PFK, PK and glycogen synthase. Its insulinomimetic action possibly mediated via Zinc-α2-glycoporteins increases cellular GLUT4 levels in skeletal muscles and adipose tissue facilitating glucose absorption. Zinc-α2-glycoprotein is gaining increasing recognition as a marker of insulin resistance in type-2 diabetes. Zinc-α2-glycoproteins are also involved in lipid metabolism, affecting the expression of several lipolytic enzymes at hepatic and adipose tissue level. Two recent meta-analyses have shown that Zinc supplementation reduces Fasting Blood Glucose, 2 h Post Prandial Blood Glucose and HbA1c in patients with diabetes, as well as reducing total cholesterol, LDL cholesterol and triglycerides in both patients with and without diabetes [15, 129]. The above molecular/enzymatic level mechanisms probably explain the beneficial effects of Zinc supplementation on glycaemic control and lipids observed in humans.

Zinc also plays an important role in the normal functioning of the islet cells of the pancreas. β-cells and their granules are extremely rich in Zinc. Zinc Transporters (ZnTs) transport zinc from the cytoplasm to extracellular spaces or to intra-cytoplasmic vacuoles, such as secretory granules, while the ZIPs are thought to increase cytoplasmic zinc [130]. Insulin production and efficient packaging into vesicles is closely linked with the transport of Zinc in to the β-cells and subsequent concentration inside vesicles mediated by the Zinc transporter ZnT8, a product of the SLC30A8 gene, specifically expressed in the β-cells of the pancreas [131]. Alterations and/or variations in the activity of ZnT8 is associated with impaired glucose induced insulin response, which promotes progression from glucose intolerance to type-2 diabetes in susceptible individuals [131]. ZnT8 has also been identified as a novel target auto-antigen in patients with type-1 diabetes and hence has diagnostic implications [132]. Auto-antibodies to ZnT8 (ZnT8A) are detected in 50–60 % of Japanese patients with acute-onset and 20 % with slow-onset type-1 diabetes [132]. Hence, it is evident that ZnT8 is a key mediator in the pathogenesis of both type-1 and type-2 diabetes. In addition to ZnT8, altered activity of many other Zinc transporters and Zinc influx proteins (ZIP 6, 7, 8) have been implicated in the pathogenesis of diabetes. Zinc is also an important mediator of α-cell function, as it inhibits glucagon secretion [93]. Identification and characterization of these ZnTs and ZIPs will help in the development of novel therapies for diabetes targeting these molecules and to develop new methods to protect β-cell mass and function, in both type-1 and type-2 diabetes.

Patients’ adherence to present therapeutic regimes for diabetes treatment are poor, resulting in unsatisfactory diabetes control [133]. Regime complexity, hypoglycaemia and other side-effects, lack of confidence in immediate or future benefits and patients’ education/beliefs are among the common reasons identified that limits adherence [134–137]. Inadequacies in current treatments has resulted in 2 to 3.6 million people in USA relying on alternative therapies for management diabetes [138]. In addition current treatment modalities are not very efficacious in preventing and/or delaying the progression of β-cell dysfunction and ultimate β-cell failure in patients with type-2 diabetes. The development of new compounds for treating diabetes is currently important to reduce the need for insulin injection in diabetic patients and to replace the clinically used synthetic therapeutics, which has several severe side effects [139].

Zinc ions and its’ numerous complexes, have shown insulin-like action both *in-vitro* and *in-vivo* [71–90]. Zinc complexes activate the insulin signaling cascade via Akt/PKB, which resultant increase in cellular GLUT4 and enhanced cellular glucose uptake. In animal models of type-2 diabetes these complexes have shown a significant ability to reduce blood glucose, HbA1c, serum insulin, triglycerides and total cholesterol, whilst improving glucose tolerance. On the basis of these findings and observations, it is evident that Zinc complexes have promise as a novel therapeutic modality that mimics the action of insulin [139]. However, presently the findings are only from *in-vitro* and *in-vivo* animal studies, there is a paucity of data from randomized controlled trials in humans. It is necessary to identify few efficacious compounds, with the least amounts of toxicity and for those complexes to be carefully evaluated in humans. The fruitful outcome of such trials may offer a novel and effective oral medication with better anti-diabetic in place of insulin [140].

## Conclusion

Numerous *in-vitro* and *in-vivo* studies have shown that Zinc has beneficial effects in both type-1 and type-2 diabetes. It is evident from the findings of the present systematic review, that Zinc plays an important role in β-cell function, insulin action, glucose homeostasis and the pathogenesis of diabetes and its complications. However further randomized double-blinded placebo-controlled clinical trials conducted for an adequate duration, are required to establish therapeutic efficacy and safety in humans.

## Additional file

Additional file 1:
**PRISMA 2009 Checklist.** (DOC 64 kb)
